# Glucose Deprivation Induces ATF4-Mediated Apoptosis through TRAIL Death Receptors

**DOI:** 10.1128/MCB.00479-16

**Published:** 2017-05-02

**Authors:** Raffaella Iurlaro, Franziska Püschel, Clara Lucía León-Annicchiarico, Hazel O'Connor, Seamus J. Martin, Daniel Palou-Gramón, Estefanía Lucendo, Cristina Muñoz-Pinedo

**Affiliations:** a Cell Death Regulation Group, Molecular Mechanisms and Experimental Therapy in Oncology Program, Bellvitge Biomedical Research Institute (IDIBELL), L'Hospitalet de Llobregat, Barcelona, Spain; b Molecular Cell Biology Laboratory, Department of Genetics, The Smurfit Institute, Trinity College, Dublin, Ireland

**Keywords:** apoptosis, ATF4, cancer metabolism, glucose, TRAIL

## Abstract

Metabolic stress occurs frequently in tumors and in normal tissues undergoing transient ischemia. Nutrient deprivation triggers, among many potential cell death-inducing pathways, an endoplasmic reticulum (ER) stress response with the induction of the integrated stress response transcription factor ATF4. However, how this results in cell death remains unknown. Here we show that glucose deprivation triggered ER stress and induced the unfolded protein response transcription factors ATF4 and CHOP. This was associated with the nontranscriptional accumulation of TRAIL receptor 1 (TRAIL-R1) (DR4) and with the ATF4-mediated, CHOP-independent induction of TRAIL-R2 (DR5), suggesting that cell death in this context may involve death receptor signaling. Consistent with this, the ablation of TRAIL-R1, TRAIL-R2, FADD, Bid, and caspase-8 attenuated cell death, although the downregulation of TRAIL did not, suggesting ligand-independent activation of TRAIL receptors. These data indicate that stress triggered by glucose deprivation promotes the ATF4-dependent upregulation of TRAIL-R2/DR5 and TRAIL receptor-mediated cell death.

## INTRODUCTION

Glucose is an essential nutrient for mammalian cells. In particular, cancer cells display glucose avidity and are more sensitive to cell death by starvation than nontransformed cells. This predisposition to cell death has been associated with the hyperactivation of oncogenes and the inactivation of tumor suppressors, most of which regulate glucose uptake and utilization (1–3).

Cell death induced by glucose deprivation is thought to occur in the nutrient-deprived necrotic core and is the desirable outcome of novel anticancer therapies that target glucose metabolism (4). However, the cell death pathways engaged by glucose deprivation remain unclear. Some reports indicate that glucose deprivation leads to energy stress or endoplasmic reticulum (ER) stress that can cause necrosis of mouse embryonic fibroblasts or rhabdomyosarcoma (5, 6). In other cell types, particularly hematopoietic tumor cells, glucose deprivation leads to mitochondrial apoptosis, which is mediated by Bcl-2 family proteins (7–9). In cells that are incapable of undergoing apoptosis through the mitochondrial pathway, such as Bax-Bak-deficient mouse embryonic fibroblasts (MEFs), glucose deprivation stimulates caspase-8-mediated apoptosis instead (10).

The signaling pathways that promote cell death in response to starvation are a subject of debate. It was thought that the loss of ATP and mTOR inactivation were the cause of cell death induced by starvation. However, it was recently shown that cell death caused by the loss of specific nutrients is a highly regulated process that depends on active signaling pathways. In response to glutamine depletion or the inhibition of glutaminolysis, the transcription factor ATF4 is induced by a mechanism involving its selective translation, and it leads to the induction of proapoptotic Bcl-2 family proteins (11). This transcription factor is part of the integrated stress response (ISR) engaged in the response to viral infection or amino acid depletion and of the unfolded protein response (UPR) activated in response to ER stress. In response to glucose deprivation or inhibition of glucose metabolism with 2-deoxyglucose, ATF4 has also been shown to be induced, and it regulates the mitochondrial apoptotic pathway (12, 13). Here we show that ATF4 is required for apoptosis induced by glucose deprivation in cells in which caspase-8 is the apoptosis-initiating caspase. Glucose deprivation induces the TRAIL receptors DR4 and DR5. TRAIL receptor 2 (TRAIL-R2)/DR5 is regulated by ATF4 in this context, and both receptors mediate cell death, with TRAIL receptor 2 being quantitatively more important.

## RESULTS

### Glucose deprivation induces apoptosis and endoplasmic reticulum stress in human tumor cells.

**FIG 1:**
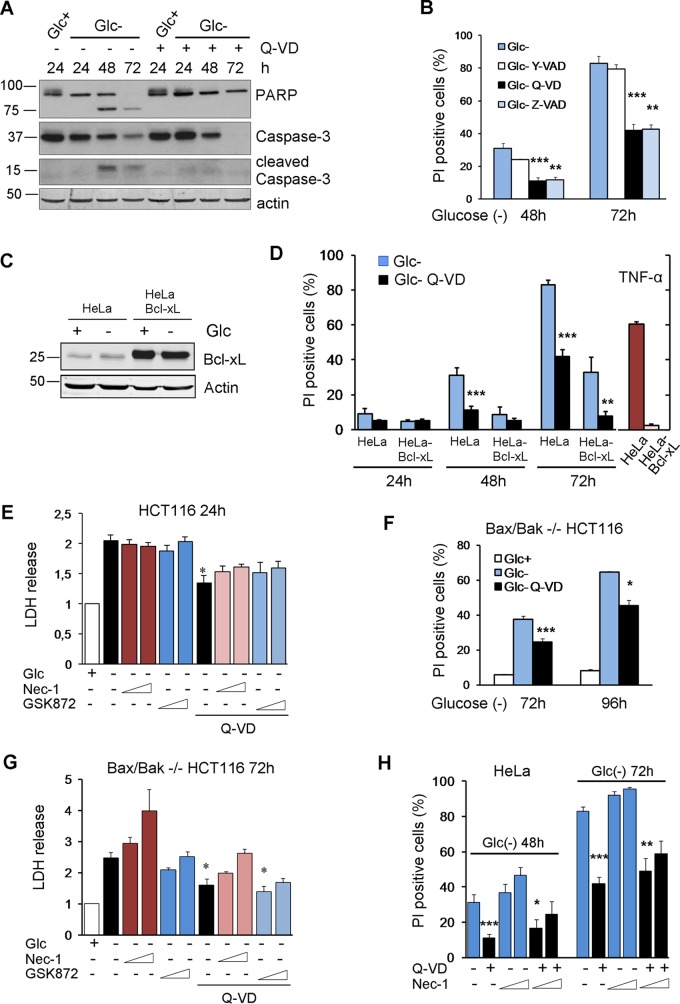
Glucose deprivation induces apoptosis and necrosis in human tumor cell lines. (A) HeLa cells were plated at 20% confluence in 60-mm dishes, and 24 h later, they were incubated with (Glc+) or without (Glc−) glucose for the indicated times in the presence of DMSO (Q-VD−) or Q-VD (Q-VD+). Western blotting of caspase-3 and PARP is shown. (B) HeLa cells were incubated in the presence or absence of glucose plus DMSO, Y-VAD, Q-VD, or Z-VAD and collected to determine PI incorporation by FACS analysis at the indicated time points. The averages and SEM of data from at least three experiments are shown. (C and D) HeLa and HeLa-Bcl-xL cells were plated at 20% confluence in 12-well plates, and 24 h later, they were incubated without glucose for 24, 48, and 72 h or with TNF plus cycloheximide for 24 h and subjected to Western blotting (C) or collected to determine PI incorporation by FACS (D). The averages and SEM of data from at least three experiments are shown. (E) HCT116 cells were incubated in the presence or absence of glucose plus DMSO, Q-VD, necrostatin-1 (40 or 100 μM), and/or a RIPK3 inhibitor (1 or 3 μM) as indicated and collected for an LDH test at 24 h. The averages and SEM of data from three experiments are shown. (F) Bax/Bak^−/−^ HCT116 cells were plated at 20% confluence in 60-mm dishes, and 24 h later, they were incubated without glucose in the presence or absence of Q-VD and collected to determine PI incorporation by FACS analysis at the indicated time points. The averages and SEM of data from three experiments are shown. (G) Bax/Bak^−/−^ HCT116 cells were incubated in the presence or absence of glucose plus DMSO, Q-VD, necrostatin-1 (40 or 100 μM), and/or a RIPK3 inhibitor (1 or 3 μM) as indicated and collected for an LDH test at 72 h. The averages and SEM of data from at least three experiments are shown. (H) HeLa cells were incubated in the absence of glucose plus DMSO, Q-VD, or necrostatin-1 or a combination of Q-VD and necrostatin-1 (1 or 3 μM) and collected for PI analysis by FACS analysis at the indicated time points. The averages and SEM of data from at least three experiments are shown. *, *P* < 0.05; **, *P* < 0.01; ***, *P* < 0.001.

Human tumor cell lines have been shown to die either by necrosis or by apoptosis upon glucose deprivation. We show here that in HeLa cells, glucose withdrawal resulted in poly(ADP-ribose) polymerase (PARP) and caspase-3 cleavage after 48 and 72 h of treatment that was prevented by cotreatment with the caspase inhibitor Q-VD (Fig. 1A), indicating that cell death is apoptosis. Moreover, HeLa cells were protected by cotreatment with the pancaspase inhibitors Q-VD and Z-VAD but not with Y-VAD, an inhibitor of caspase-1, indicating that cell death is due to apoptotic and not inflammatory caspases (Fig. 1B). To test whether these cells died through mitochondrial apoptosis, we subjected HeLa cells overexpressing Bcl-xL (14) to glucose deprivation and observed that they also died in a caspase-dependent manner, although they were partially protected, and thus, the kinetics were slower (Fig. 1C and D). These cells were completely protected from tumor necrosis factor (TNF), which kills HeLa cells in a mitochondrion-dependent manner. Additionally, we subjected HCT116 cells and their Bax-Bak-deficient counterparts to glucose deprivation and observed that they died with different kinetics, but in both cases, they were significantly protected by cotreatment with Q-VD (Fig. 1E to G). In all cell lines, a necrotic component was observed at later time points. Since HeLa cells do not express RIPK3 (15), this component is probably not necroptotic. However, we employed inhibitors of RIPK1 and RIPK3 in HCT116 cells and their Bax-Bak-deficient counterparts and observed that these inhibitors do not confer protection from cell death when incubated in the presence or absence of the caspase inhibitor Q-VD (Fig. 1F and G). Similarly, HeLa cells were not protected by using necrostatin-1 (Fig. 1H). Indeed, this compound was slightly toxic at high concentrations and longer times, possibly as an inhibitor of the tryptophan-catabolizing enzyme IDO (indoleamine-pyrrole 2,3-dioxygenase) (Fig. 1F to H) (16). All these data suggested that glucose deprivation activates apoptosis partially independently of the mitochondrial apoptotic pathway, together with nonnecroptotic necrosis.

**FIG 2:**
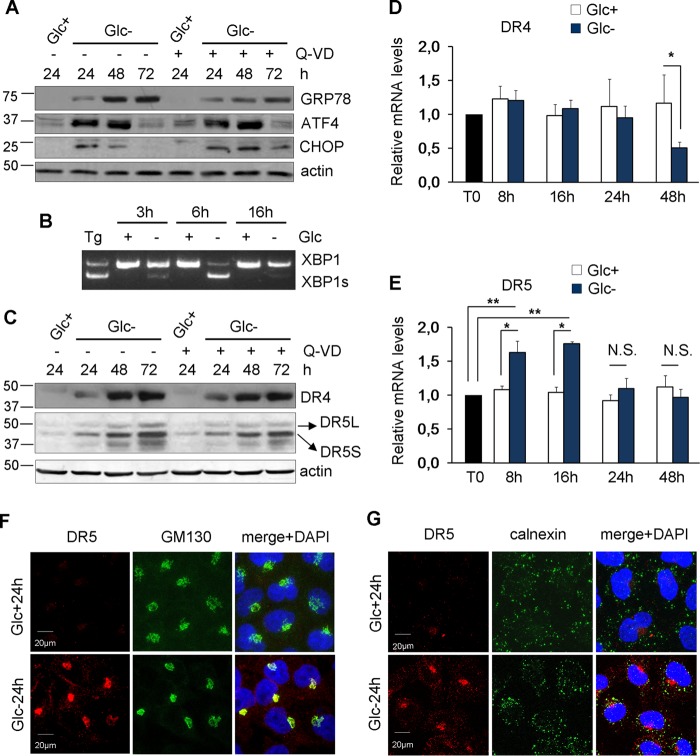
Glucose deprivation induces ER stress and TRAIL receptors. (A) HeLa cells were incubated with glucose and/or Q-VD (Q-VD+) or DMSO (Q-VD−) as indicated for 24, 48, and 72 h and collected for Western blotting of GRP78, ATF4, and CHOP. (B) HeLa cells were treated with thapsigargin (Tg) for 24 h or incubated in the presence (+) or absence (−) of glucose for the indicated times and collected for reverse transcription-PCR analysis of unspliced and spliced XBP1. (C) HeLa cells were incubated with glucose and/or Q-VD (Q-VD+) or DMSO (Q-VD−) as shown for the indicated times and then collected for Western blotting of DR4 (TRAIL-R1) and DR5 (TRAIL-R2). (D and E) HeLa cells were incubated with or without glucose for the times shown and collected for qPCR analysis. DR4 mRNA levels relative to the values for the housekeeping gene and time zero (T0) are reported in panel B. DR5 mRNA levels relative to the values for the housekeeping gene and time zero (T0) are reported in panel C. The averages and SEM of data from at least three experiments are shown. (F and G) HeLa cells were plated for immunofluorescence, and 24 h later, they were incubated with or without glucose for 24 h before performing confocal analysis of DR5 and GM130 (F) or calnexin (G) localization. *, *P* < 0.05; **, *P* < 0.01; N.S., not significant.

We and others have shown that the transcription factor ATF4 plays a role in cell death induced by the deprivation of glucose or glutamine (6, 11, 17) or by treatment with the nonmetabolizable glucose analog 2-deoxyglucose (12). ATF4 is central to the integrated stress response and the unfolded protein response induced, among other stimuli, upon ER stress. Glucose is required to provide glycosylation precursors, and its absence disturbs the ER and Golgi apparatus (18). Additionally, glucose deprivation may cause a secondary loss of amino acids, which is known to activate the ISR, which is mediated by the uncharged tRNA-activated kinase GCN2. As shown in Fig. 2A and B, glucose deprivation induces ATF4, its downstream effector CHOP, and ER stress, as measured by the induction of the chaperone GRP78/Bip and splicing of the mRNA of XBP1, a target of the ER stress sensor IRE1. This suggests that the ISR/UPR may participate in cell death, as previously observed for other cell lines (19).

### Glucose deprivation regulates levels of TRAIL receptors.

Stimuli that promote endoplasmic reticulum stress, such as tunicamycin and thapsigargin, can induce apoptotic cell death through the mitochondrial pathway. However, they have also been shown to induce TRAIL receptors and to be sensitized to TRAIL (20). Intriguingly, some TRAIL receptors can mediate ER stress-induced cell death in a manner independent of death receptor-death ligand interactions (21–23). Since glucose deprivation induced ATF4 and CHOP, the latter of which has been linked to TRAIL receptor transcription (24), we examined the levels of TRAIL receptors upon treatment without glucose. Figure 2C shows that glucose deprivation strongly induced the accumulation of the TRAIL receptors DR4/TRAIL-R1 and DR5/TRAIL-R2. Next, we analyzed their mRNA levels. Quantitative PCR (qPCR) analysis indicated that mRNA levels of *DR4* did not change upon glucose withdrawal at any time examined, with the exception of later time points at which *DR4* mRNA levels are reduced (Fig. 2D). In contrast, the accumulation of DR5 may involve its transcriptional upregulation at short times (Fig. 2E), although by 24 h of glucose deprivation, *DR5* mRNA levels returned to control levels, while the protein levels continued to increase. DR5 was previously shown to be localized mainly in intracellular membranes upon treatment with ER-stressing drugs (22), although an increase in plasma membrane translocation was also detected (25). We analyzed DR5 localization in cells grown without glucose and observed that the majority of DR5 detected after treatment accumulated in the Golgi apparatus and did not colocalize with mitochondria or the endoplasmic reticulum protein calnexin (see Fig. 6F and G and data not shown). However, we also observed some accumulation in the plasma membrane (see Fig. 6F and G).

Altogether, these data indicated that glucose deprivation regulates the expression of TRAIL receptors, possibly via ER stress-dependent pathways and most likely in a posttranscriptional manner.

### Caspase-8, FADD, and Bid mediate apoptosis induced by glucose deprivation.

**FIG 3:**
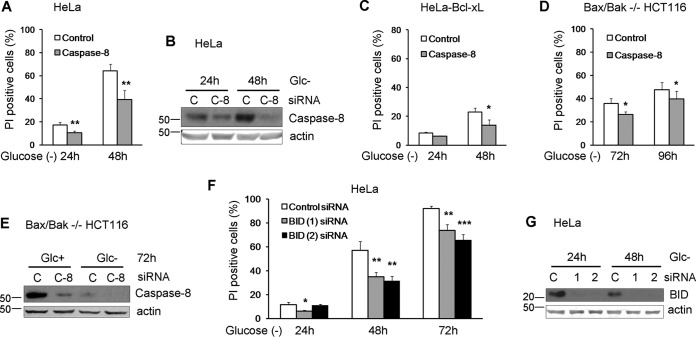
Human cell lines die in a caspase-8- and Bid-dependent manner under glucose deprivation. (A to C) HeLa cells (A and B) and HeLa-Bcl-xL cells (C) were plated at 50% confluence in 6-well plates, and 5 h later, they were transfected with caspase-8 or control siRNA. At 24 h posttransfection, cells were treated without glucose for 24 and 48 h and collected to determine PI incorporation by FACS analysis. The averages and SEM of data from three experiments are shown. Western blotting of control and caspase-8 siRNA-transfected HeLa cells treated without glucose for the indicated times is shown in panel B. (D and E) Bax/Bak^−/−^ HCT116 cells were plated at 50% confluence in 6-well plates, and 5 h later, they were transfected with caspase-8 siRNA or control siRNA. At 24 h posttransfection, cells were incubated without glucose for 72 and 96 h and collected to determine PI incorporation by FACS analysis. The averages and SEM of data from three experiments are shown. Western blotting of cells transfected with control or caspase-8 siRNAs is shown in panel E. (F and G) HeLa cells were plated at 50% confluence in 6-well plates, and 5 h later, they were transfected with Bid (1) and Bid (2) siRNAs or the control siRNA. At 24 h posttransfection, cells were treated without glucose for 24, 48, and 72 h and collected to determine PI incorporation by FACS analysis. The averages and SEM of data from three experiments are shown. Western blotting of cells transfected with control, Bid (1), or Bid (2) siRNAs and treated without glucose for the indicated times is shown in panel G. *, *P* < 0.05; **, *P* < 0.01; ***, *P* < 0.001.

Caspase-8 is the apical caspase in the death receptor pathway, which cleaves and activates executioner caspases directly or indirectly upon death receptor ligation. Death receptors activate caspase-8 through the recruitment of adapter molecules to a death-inducing signaling complex (DISC) to which this protease is recruited. Previously, we showed that Bax-Bak-deficient MEFs subjected to glucose deprivation die in a caspase-8-dependent manner that could bypass the mitochondrial pathway (10). To test whether caspase-8 was involved in the apoptosis of HeLa cells, we silenced caspase-8 using small interfering RNA (siRNA), and we detected a significant reduction of cell death under glucose deprivation (10) (Fig. 3A and B). siRNA against this protease revealed that caspase-8 also participated in the apoptosis of Bcl-xL-overexpressing HeLa cells (Fig. 3C). Moreover, the apoptosis of Bax-Bak-deficient HCT116 cells was also dependent on caspase-8, although the amount of protection conferred by the siRNA was small, consistent with the small amount of protection conferred by caspase inhibitors (Fig. 1D and 3D and E). All these data suggest that glucose deprivation induces caspase-8-dependent apoptosis in diverse human tumor lines with either an intact or a deficient mitochondrial pathway.

**FIG 4:**
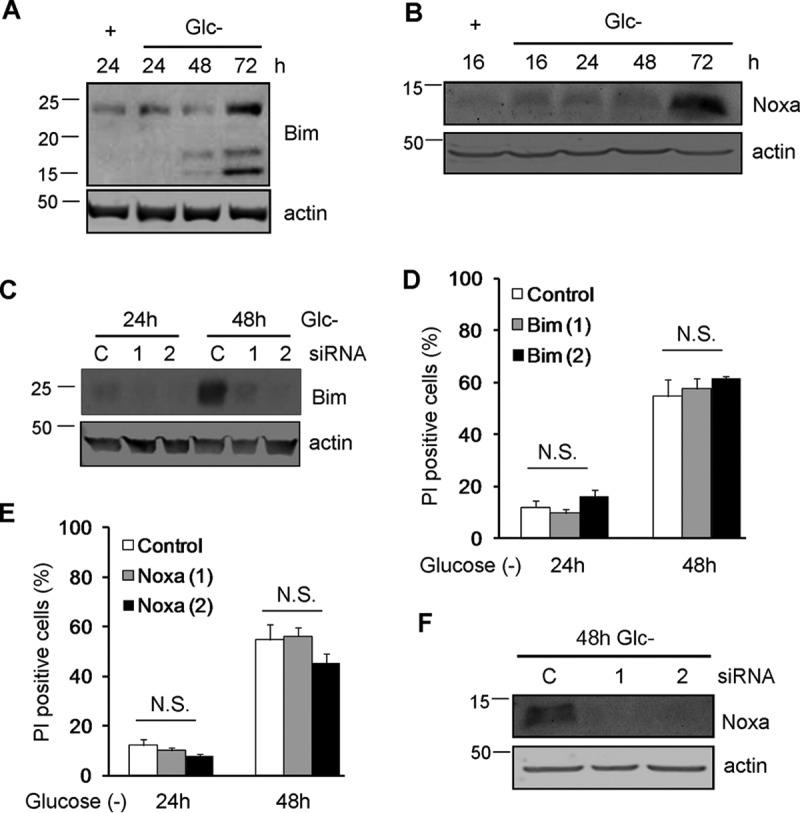
Noxa and Bim are dispensable for glucose deprivation-induced cell death of HeLa cells. (A and B) HeLa cells were incubated in the presence (+) or absence (Glc−) of glucose for the indicated times and collected for Western blotting of Bim (A) and Noxa (B). (C and D) HeLa cells were transfected with control siRNA or siRNAs against Bim, incubated for 24 and 48 h without glucose, and collected for Western blotting of Bim (C) or FACS analysis (D). (E and F) HeLa cells were transfected with siRNA against Noxa, incubated for the indicated times without glucose, and collected for FACS analysis (E) and Western blot analysis (F). N.S., not significant.

The fact that HeLa cells overexpressing Bcl-xL died slower suggested that the mitochondria could participate in apoptosis in these cells through the cleavage of Bid by caspase-8. Indeed, silencing of Bid in HeLa cells partially prevented cell death (Fig. 3F and G). In contrast, levels of BH3-only proteins such as Bim and Noxa, previously linked to apoptosis induced by ER stress or by starvation (reviewed in reference 21), were increased with treatment (Fig. 4A and B), but they did not participate in cell death, as revealed by siRNA (Fig. 4C to F).

**FIG 5:**
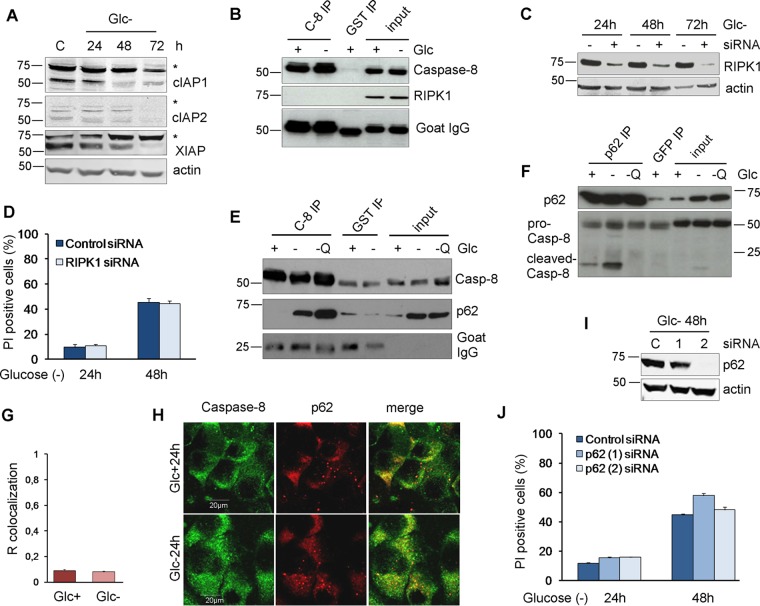
RIPK1 and p62 are not involved in cell death under glucose deprivation. (A) HeLa cells were plated at 20% confluence in 60-mm dishes, and 24 h later, they were incubated without glucose for 24, 48, and 72 h and collected for Western blot analysis of cIAPs and XIAP. (B) HeLa cells were plated at 20% confluence in 100-mm dishes, and 24 h later, they were incubated with or without glucose for 24 h before performing immunoprecipitation of caspase-8 or GST as a control. Western blots of caspase-8 and RIPK1 are shown. (C) Western blot analysis of glucose-deprived cells transfected with control (−) or RIPK1 (+) siRNA and incubated without glucose for the times shown. (D) HeLa cells were plated at 50% confluence in 6-well plates, and 5 h later, they were transfected with 50 nM RIPK1 siRNAs or the control siRNA. Twenty-four hours after transfection, cells were incubated without glucose for 24 and 48 h and collected for PI analysis by FACS analysis. The averages and SEM of data from three experiments are shown. (E and F) HeLa cells were plated at 20% confluence in 100-mm dishes, and 24 h later, they were incubated with glucose (+), without glucose or Q-VD (−), or without glucose but with 10 μM Q-VD (-Q) for 24 h before immunoprecipitation of caspase-8 (E) or p62 (F) was performed. Western blot analyses of caspase-8 and p62 are shown. (G and H) HeLa cells were plated for immunofluorescence analysis, and they were treated with or without glucose for 24 h. Representative confocal microscopy images of caspase-8 and p62 (H) and data from colocalization analysis using ImageJ software (G) are shown. The graph shows the averages and the standard deviations of data from four independent experiments. (I and J) HeLa cells were transfected with 50 nM p62 (1) and p62 (2) siRNAs and the control siRNA. At 24 h posttransfection, cells were incubated without glucose for 24 and 48 h and collected to determine PI incorporation by FACS analysis. Western blotting of p62 is shown in panel I. The averages and SEM of data from three experiments are shown in panel J. Differences between siRNAs were not statistically significant.

Caspase-8 is usually activated upon its dimerization in intracellular or membrane DISCs through recruitment by the adapter molecule FADD in response to the interaction of death ligands with death receptors. We previously analyzed the role of these interactions in cell death induced by glucose deprivation in Bax-Bak-deficient MEFs and could not detect any role of death ligand-receptor interactions (10). For this reason, we analyzed two unconventional platforms to activate caspase-8: the ripoptosome and p62. The ripoptosome is nucleated by the kinase RIPK1 and is formed upon the inhibition and degradation of cellular inhibitor of apoptosis proteins (cIAP) (26, 27). We observed that glucose deprivation promoted the disappearance of cIAP1, cIAP2, and X-linked inhibitor of apoptosis protein (XIAP) (Fig. 5A). This, however, did not lead to a detectable interaction of RIPK1 and caspase-8 in the absence (Fig. 5B) or presence (data not shown) of caspase inhibitors. Moreover, the downregulation of RIPK1 did not prevent cell death induced by glucose deprivation (Fig. 5C and D). Caspase-8 has been shown to interact with the autophagosomal proteins LC3 and p62 upon several stimuli, with p62 contributing to its aggregation and activation (21, 28, 29). We previously described the accumulation of p62 upon glucose deprivation due to an inactivation of autophagic flux (30) (Fig. 5E and F, input). We observed that caspase-8 coimmunoprecipitated with p62 upon glucose deprivation (Fig. 5E and F), However, there was no measurable translocation of caspase-8 to p62 spots (aggresomes) (Fig. 5G and H), and similar to what we previously observed in Bax-Bak-deficient MEFs (30), p62 knockdown did not prevent glucose deprivation-induced cell death (Fig. 5I and J).

**FIG 6:**
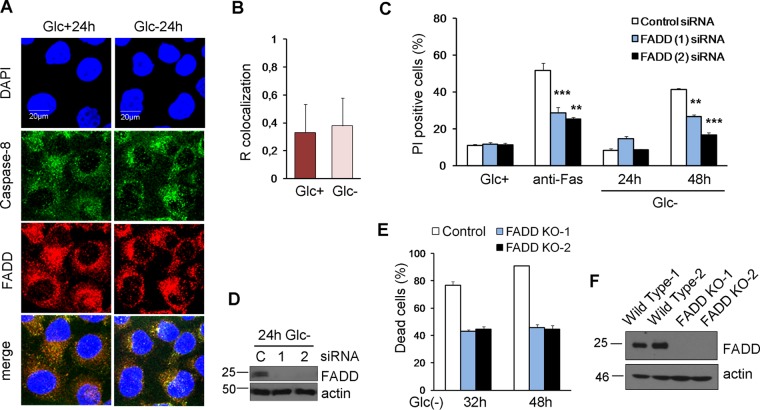
FADD is involved in cell death under glucose deprivation. (A and B) HeLa cells were plated for immunofluorescence, and 24 h later, they were incubated with (Glc+) or without (Glc−) glucose for 24 h. Confocal microscope pictures of colocalization of caspase-8 with FADD (A) and quantification by using ImageJ software (B) are reported. The graph shows the averages and the standard deviations of data from four independent experiments. (C and D) HeLa cells were transfected with control, FADD (1), or FADD (2) siRNA. At 48 h posttransfection, cells were incubated without glucose for 24 and 48 h or with an anti-Fas antibody for 48 h and collected for Western blotting (D) and for determining PI incorporation by FACS analysis (C). The averages and SEM of data from at least three experiments are shown. (E and F) HeLa cells knocked out for FADD (clones 1 and 2) and their controls were subjected to glucose deprivation for the indicated times and subjected to Western blot analysis (F) or determination of the number of dead cells under a microscope (E). Data show the averages and SEM of results from three independent experiments. **, *P* < 0.01; ***, *P* < 0.001.

In order to examine the role of FADD, we performed colocalization analysis of caspase-8 and FADD by immunostaining under normal and glucose-deprived conditions (Fig. 6A and B). We detected a basal interaction of the two proteins, but no changes were observed after glucose removal. In order to clarify the role of FADD in cell death induced by glucose deprivation, we employed siRNA to target this protein (Fig. 6C) and observed that its downregulation protected from glucose deprivation to the same extent that it protected from cross-linking the Fas receptor with an antibody (Fig. 6D). Moreover, the elimination of FADD by using clustered regularly interspaced short palindromic repeats (CRISPR) prevented cell death by glucose deprivation (Fig. 6E and F).

### ATF4 and TRAIL receptors mediate apoptosis upon glucose deprivation.

**FIG 7:**
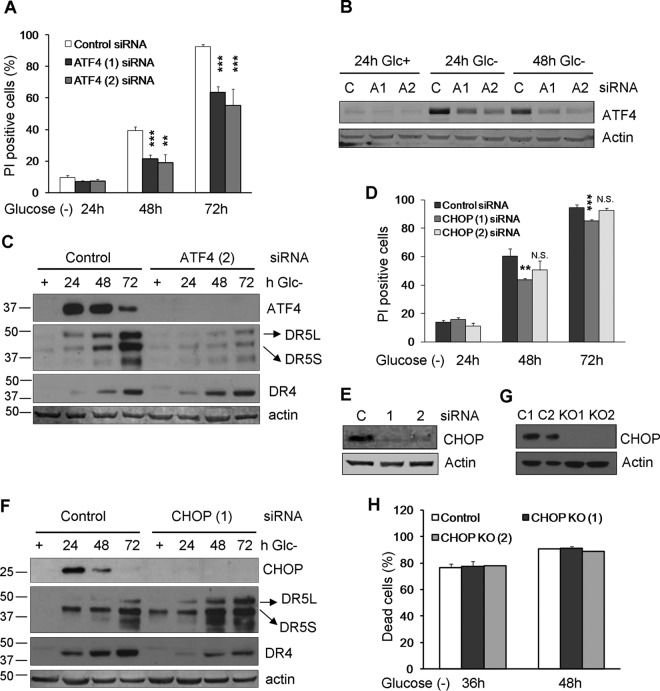
ATF4 and CHOP. (A to C) HeLa cells were transfected with ATF4 siRNAs or the control siRNA. At 24 h posttransfection, cells were incubated without glucose for 24, 48, and 72 h and collected to determine PI incorporation by FACS analysis (C). The averages and SEM of data from four to seven experiments under each condition are shown. Western blots of ATF4, DR5, and DR4 in cells transfected with control, ATF4 (1), and ATF (2) siRNAs are shown in panels B and C. (D to F) HeLa cells were transfected with CHOP siRNAs and the control siRNA. At 24 h posttransfection, cells were incubated without glucose for 24, 48, and 72 h and collected to determine PI incorporation by FACS analysis (D). The averages and SEM of data from at least three experiments are shown. Western blots of CHOP, DR5, and DR4 in cells transfected with control or CHOP siRNAs are shown in panels E and F. (G and H) HeLa cells knocked out for CHOP (clones 1 and 2) and their controls were subjected to glucose deprivation for the indicated times and subjected to Western blot analysis after treatment with 10 μg/ml brefeldin A or determination of the number dead cells under a microscope. Data show averages and SEM of results from three independent experiments. **, *P* < 0.01; ***, *P* < 0.001; N.S., not significant.

It has been reported that ER stressors induce DR4 and DR5 through the transcription factor CHOP, which is itself a transcriptional target of ATF4 (24, 31). We thus investigated a role for the ATF4/CHOP axis in apoptosis induced by glucose deprivation and in the accumulation of death receptors. ATF4 downregulation with siRNA strongly reduced cell death, as described by other models of starvation (Fig. 7A and B). Its downregulation also prevented DR5 accumulation although not DR4 expression (Fig. 7C). We analyzed the role of CHOP and observed that different siRNAs partially protected from cell death (Fig. 7D and E). Different from a number of studies that reported the induction of DR5 mediated by CHOP in response to other stimuli, siRNA against CHOP did not prevent DR5 induction induced by glucose deprivation (Fig. 7F). However, a modest reduction of the accumulation of DR4 was observed. Because the amount of protection from cell death conferred by siRNA was very small, we generated cells lacking CHOP via CRISPR knockout (Fig. 7G and H). These cells showed no protection from cell death, which indicates that cell death is mediated by ATF4 but is independent of CHOP.

**FIG 8:**
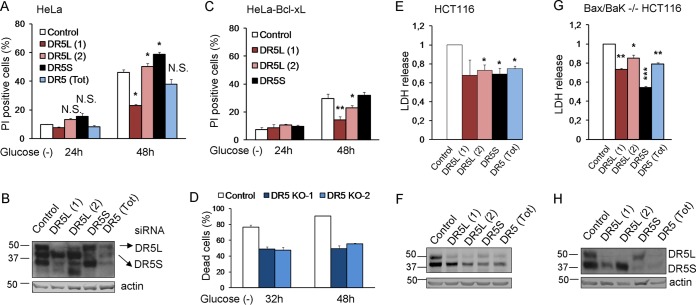
DR5 siRNAs protect from death induced by glucose deprivation. (A and B) HeLa cells were transfected with DR5L (1), DR5L (2), DR5S, DR5 (Tot), or the control siRNA. At 24 h posttransfection, cells were incubated without glucose for 24 and 48 h and collected to determine PI incorporation by FACS analysis (A) or Western blot analysis of DR5 48 h after treatment (B). The averages and SEM of data from at least three experiments are shown. (C) HeLa-Bcl-xL cells were transfected with DR5L (1), DR5L (2), DR5S, or the control siRNA. At 24 h posttransfection, cells were incubated without glucose for 24 and 48 h and collected to determine PI incorporation by FACS analysis. The averages and SEM of data from at least three experiments are shown. (D) Control HeLa cells and cells deficient in *DR5* (KO1 and KO2) were incubated without glucose for the indicated times, and the number of dead cells was determined with a microscope. Shown are averages and SEM of data from three independent experiments. Western blotting for the expression of DR5 is shown in Fig. 9F. (E and F) HCT116 cells were transfected with DR5L (1), DR5L (2), DR5S, DR5 (Tot), or the control siRNA. At 24 h posttransfection, the cells were incubated without glucose for 24 h and collected for an LDH test. The averages and SEM of data from four experiments are shown. Western blotting of DR5 after 24 h of treatment is shown in panel F. (G and H) Bax/Bak^−/−^ HCT116 cells were transfected with DR5L (1), DR5L (2), DR5S, DR5 (Tot), or the control siRNA. At 24 h posttransfection, cells were incubated without glucose for 72 h and collected for an LDH test. The averages and SEM of data from four experiments are shown. Western blotting of DR5 in Bax/Bak^−/−^ HCT116 cells 72 h after treatment is shown in panel H. *, *P* < 0.05; **, *P* < 0.01; ***, *P* < 0.001; N.S., not significant.

We subsequently analyzed the role of TRAIL death receptors in apoptosis induced by glucose deprivation. siRNAs against DR5 protected from cell death, although protection was not uniform, and their strength depended on the sequence employed (Fig. 8A and B). Up to five bands corresponding to different isoforms of DR5 (long and short) and possibly their unglycosylated counterparts were detected upon glucose deprivation (Fig. 8B). It was previously shown that DR5-mediated cell death in response to thapsigargin is dependent on the long isoform of DR5 (22). We observed that siRNA against the short isoform (DR5S) did not prevent the death of HeLa cells subjected to glucose deprivation (Fig. 6A and B). In contrast, sequences that downregulated the long isoform (DR5L) were effective in protecting HeLa and HeLa-Bcl-xL cells from apoptosis, correlating with their efficiency in suppressing DR5L expression (Fig. 8A to C). In order to verify the results obtained by using siRNA, we generated cells deficient in DR5, and we observed protection from cell death (Fig. 8D). DR5 downregulation also prevented the death of HCT116 and Bax-Bak-deficient HCT116 cells, and in these cells, the downregulation of the short isoform of DR5 was also effective (Fig. 8E to H).

**FIG 9:**
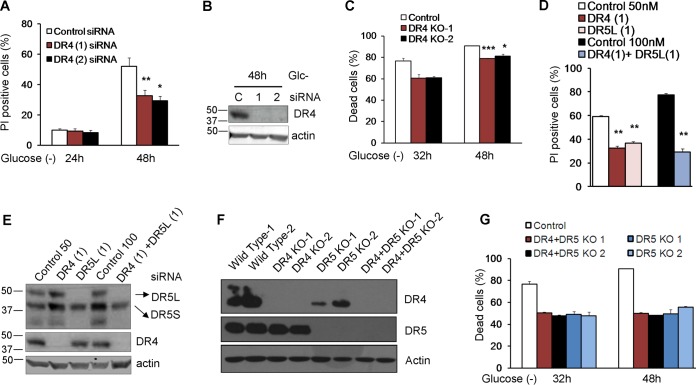
DR4 is involved in cell death under glucose deprivation. (A and B) HeLa cells were transfected with DR4 (1), DR4 (2), or the control siRNA. At 24 h posttransfection, cells were incubated without glucose for 24 and 48 h and collected to determine PI incorporation by FACS analysis. The averages and SEM of data from at least three experiments are shown in panel A. Western blotting of cells treated for 48 h without glucose and transfected with control, DR4 (1), or DR4 (2) siRNA is shown in panel B. (C) Control HeLa cells and cells deficient in *DR4* (KO1 and KO2) were incubated without glucose for the indicated times, and the dead cells were counted under a microscope. Shown are averages and SEM of data from three independent experiments. Western blotting is shown in panel F. (D) HeLa cells were transfected with DR4 (1), DR5L (1), or control siRNA (50 nM) or the combination of DR4 (1) plus DR5L (1) siRNAs at 100 nM as a control. At 24 h posttransfection, cells were incubated without glucose for 24 and 48 h and collected to determine PI incorporation by FACS analysis. The averages and SEM of data from at least three experiments are shown. Numbers indicate the average protection (percent) conferred by each siRNA or siRNA combination versus its control. (E) Western blotting of DR5 and DR4 in HeLa cells 48 h after treatment. (F) Western blotting of knockout cells (*DR4*, *DR5*, and *DR4-DR5* double knockout) generated as described in Materials and Methods. (G) Control HeLa cells and cells doubly deficient in *DR4* and *DR5* (KO1 and KO2) were incubated without glucose for the indicated times, and the dead cells were counted under a microscope. Shown are averages and SEM of data from three independent experiments and include *DR5* data from Fig. 8D for comparison. Western blotting is shown in panel F. *, *P* < 0.05; **, *P* < 0.01; ***, *P* < 0.001.

Since we also observed an upregulation of DR4, we employed siRNAs against this TRAIL receptor and observed that they produced a small but significant reduction of cell death in HeLa cells (Fig. 9A and B). The deletion of *DR4* via CRISPR significantly protected HeLa cells from glucose deprivation (Fig. 9C). We also observed that the combination of DR4 and DR5 siRNAs conferred better protection from glucose deprivation than did the single siRNAs (*P* = 0.04 for DR4 versus the combination; *P* = 0.01 for DR5 versus the combination) (Fig. 9D and E). However, the double combination of CRISPR-deleted DR4 and DR5 did not provide significantly better protection than did DR5 alone (Fig. 9F and G).

**FIG 10:**
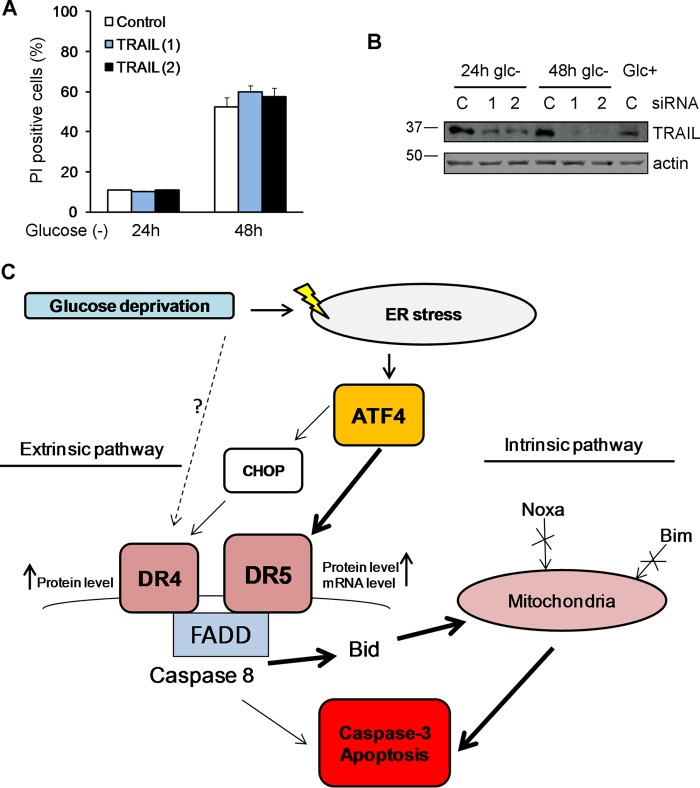
TRAIL does not participate in cell death. HeLa cells were transfected with 50 nM TRAIL siRNAs or the control siRNA. At 24 h posttransfection, cells were incubated with or without glucose for 24 and 48 h. (A) Averages and SEM of data from three experiments to determine PI incorporation by FACS analysis. (B) Western blotting of cells incubated for 24 h or 48 h without glucose or for 24 h with glucose and transfected with control, TRAIL (1), or TRAIL (2) siRNA. (C) Summary of pathways leading to cell death of glucose-deprived HeLa cells.

DR5 has been shown to play a role in cell death induced by other stimuli that induce ER stress: thapsigargin and the GRP78-depleting bacterial agent subtilase cytotoxin AB (22, 23). Moreover, the authors of that study reported that TRAIL receptor-mediated cell death occurred in a manner independent of the ligand TRAIL upon treatment with ER inducers. We tested whether TRAIL participated in cell death induced by glucose deprivation in these cells. In agreement with our previous results in which a blockade of death receptor-ligand interactions did not prevent cell death by glucose deprivation in MEFs (10), the downregulation of TRAIL using two different siRNA oligonucleotides did not prevent cell death (Fig. 10A and B).

## DISCUSSION

This study places the integrated stress response and ER stress-associated factor ATF4 as a central player in apoptosis induced by glucose deprivation. ATF4 is one of the small subsets of proteins induced under conditions of general translational repression upon the phosphorylation of the translation initiation factor eIF2α. At least two kinases are responsible for eIF2α phosphorylation upon glucose deprivation: PERK and GCN2 (19, 32). PERK participates in the unfolded protein response and is activated by endoplasmic reticulum stress, an event that occurs when the glucose level is low, probably due to the accumulation of misfolded, unglycosylated proteins. GCN2 is activated in the ISR by amino acid loss (11). The role of these responses and the subsequent translational arrest is to restore homeostasis; however, eIF2α phosphorylation and ATF4 induction also induce cell death if damage is not repaired. The role of ATF4 in apoptosis induced by “classical” ER stressors has been widely documented, although it is unclear which proteins mediate cell death in this context, and it may depend on the cell type or the coactivation of other pathways (21). Only recently has this protein emerged as a crucial regulator of metabolic sensing and also as a cell death effector upon amino acid depletion (11, 17).

We previously reported that ATF4 participates in mitochondrial apoptosis induced by the glucose analog 2-deoxyglucose, which works as an ER stressor (12). In addition, it was recently shown that ATF4 participates in the induction of apoptosis in response to glucose deprivation in HEK293 cells (13) and in the necrosis of rhabdomyosarcoma cells deprived of glucose (6). However, in these studies, the mechanism of cell death was not elucidated. Shin et al. reported a role for the BH3-only protein Bid downstream of ATF4 (13). Our data indicate that Bid, Bax, and Bak participate in the transduction of cell death signals from caspase-8 to mitochondria, although apoptosis also occurred in Bcl-xL-overexpressing or Bax-Bak-deficient cells. Cell death in response to ER stressors, including glucose deprivation and inhibitors of glycolysis, is generally thought to be mediated through the transcriptional activation of the mitochondrial pathway of apoptosis by CHOP downstream of ATF4. Indeed, CHOP induces Bim in response to the glycolytic inhibitor 2-deoxyglucose (33). CHOP is not only important for the activation of the mitochondrial pathway but is also the ER stress-associated transcription factor that mediates TRAIL receptor induction in several cell lines upon ER stress (22–24, 31). However, ER stress-dependent, CHOP-independent upregulation of DR5 has also been documented (20). In the present study, and in contrast to other reports, CHOP did not play a role in the induction of DR5, although participation in DR4 accumulation upon glucose deprivation was observed. However, the mRNA levels of *DR4* do not change after the treatment, which suggests that the regulation of this receptor is achieved mostly at the posttranscriptional level. In contrast, we observed the transcriptional induction of DR5 at early time points of glucose removal and a strong accumulation of the protein at later time points, which could be prevented by ATF4 but not CHOP silencing. These data suggest that ATF4 might directly regulate the expression of DR5, independently of CHOP, possibly at both the transcriptional and posttranscriptional levels (Fig. 10).

Downstream of death receptors, glucose deprivation has been described to reduce FLIP levels, thus leading to sensitization to TRAIL, although FLIP downregulation does not occur in all cell types (34, 35). We describe here an unequivocal role of FADD as a mediator of apoptosis induced by glucose deprivation, while the FADD- and caspase-8-interacting protein RIPK1 did not play any role in cell death, nor did p62, which we have previously shown accumulates upon glucose deprivation in these cells and can regulate caspase-8 activation downstream of TRAIL receptors (28, 30). A possible role of TRADD and other adapter molecules remains to be tested.

DR5 has been shown in several reports to play a role in cell death induced by other stimuli that induce ER stress, most notably thapsigargin (22–24). The role of DR4 is less established, although it has also been documented (31). The precise mechanism that activates these receptors upon glucose deprivation remains to be determined. In the case of “classical” ER stressors such as tunicamycin and thapsigargin, death mediated by DR5 could not be blocked by using siRNA against TRAIL or incubating cells in the presence of an extracellular, soluble form of TRAIL receptors (22, 23). Here we show that the downregulation of TRAIL to undetectable levels did not prevent cell death by glucose deprivation. This suggests that these receptors are activated through ligand-independent, intracellular aggregation. In fact, it is known that the overexpression of death receptors can induce their activation (36). This aggregation could possibly occur in membranes of the secretory pathway, such as those of the Golgi apparatus, although this remains to be proven, and the mechanism remains to be identified. Glucose is required not only for energy production but also as a substrate for the posttranscriptional modification of proteins. It is possible that the lack of glucose modifies trafficking through secretory pathways, which, together with the modification of glycosylation patterns of the receptors themselves, may promote ligand-independent associations.

## MATERIALS AND METHODS

### Cell culture and treatments.

HeLa cells from the ATCC and Bax/Bak^−/−^ simian virus 40 (SV40)-transformed MEFs (37) were cultured in pyruvate-free high-glucose (25 mM) DMEM (Dulbecco's modified Eagle's medium; Gibco Life Technologies, Waltham, MA). HCT116 cells and Bax/Bak^−/−^ HCT116 cells (provided by M. Rehm, Royal College of Surgeons Ireland) were cultured in RPMI medium supplemented with 10% FBS (fetal bovine serum; Invitrogen, Carlsbad, CA), 200 mg/ml of penicillin (Invitrogen), 100 mg/ml of streptomycin (Invitrogen), and 2 mM glutamine (Invitrogen). Cells were maintained at 37°C in a 5% CO_2_ atmosphere and split 3 times per week by using a 0.05% trypsin–EDTA solution (Invitrogen). For glucose deprivation treatment, cells were plated at a concentration of 150,000 cells/ml in 6-well plates or 60-mm dishes (BD) and treated 24 h later, when they reached a concentration of 500,000 cells/ml, corresponding to 80% confluence. Cells were washed twice with FBS-free, pyruvate-free, glucose-free DMEM (Gibco Life Technologies) and then treated with this medium supplemented with 200 mg/ml penicillin, 100 mg/ml streptomycin, freshly added 2 mM glutamine, and 10% dialyzed FBS. The same procedure was used for treating Bax/Bak^−/−^ HCT116 cells by using glucose-free RPMI medium (Gibco Life Technologies). Q-VD-OPH (apeXbio, Houston, TX) and Ac-Y-VAD-cmk (Sigma-Aldrich, St. Louis, MO) were used at 10 μM, Z-VAD-fmk (apeXbio) was used at 20 μM, and all components were added at the moment of treatment. The same amount of dimethyl sulfoxide (DMSO) was added to control wells. TNF-α (Peprotech, Le-Perray-en-Yvelines, France) was used at 10 ng/ml in combination with 10 μM cycloheximide. Necrostatin-1 (catalog number BML-AP309-0020; Enzo, Farmingdale, NY) was used at 40 and 100 μM, while a RIPK3 inhibitor (GSK'872; Calbiochem, Darmstadt, Germany) was used at 1 and 3 μM. Fas human-activating CH11 antibody (Millipore, Darmstadt, Germany) was used at 50 ng/ml. Thapsigargin (Sigma-Aldrich) was used at 300 ng/ml.

### siRNA transfection.

HeLa and Bax/Bak^−/−^ HCT116 cells were plated into 6-well plates at a confluence of 50% and transfected 5 h later. A transfecting solution containing 50 nM each siRNA and 1 μl/ml Dharmafect1 (Fisher-Thermo Scientific, Waltham, MA) was incubated for 30 min in DMEM without serum and antibiotics and then added to the cells that had previously been incubated in DMEM without antibiotics. Cells were maintained in transfection medium for 24 h or 48 h in the case of FADD siRNAs before treatments were performed. Control sequences were a nontargeting sequence (5′-UAAGGCUAUGAGAGAUAC-3′) and an On-Target control (Dharmacon, Lafayette, CO). siRNA sequences were 5′-GCCUAGGUCUCUUAGAUGA-3′ for ATF4 (1), 5′-CCAGAUCAUUCCUUUAGUUUA-3′ for ATF4 (2), 5′-GAAGACAUCAUCCGGAAUA-3′ for Bid (1), 5′-CAGGGATGAGTGCATCACAAA-3′ for Bid (2), 5′-GCACCCAUGAGUUGUGACA-3′ for Bim (1), 5′-GACCGAGAAGGUAGACAAUUG-3′ for Bim (2), Dharmacon On-Target Plus siRNA pools of 4 oligonucleotides for caspase-8 in HCT116 cells, 5′-GGAGCUGCUCUUCCGAAUU-3′ for caspase-8 in HeLa cells, 5′-AAGAACCAGCAGAGGUCACAA-3′ for CHOP (1), 5′-GCCTGGTATGAGGACCTGC for CHOP(2), 5′-CACCAAUGCUUCCAACAAU-3′ for DR4 (1), 5′-AACGAGAUUCUGAGCAACGCA-3′ for DR4 (2), 5′-GACCCUUGUGCUCGUUGUC-3′ for DR5 (Tot), 5′-CCUGUUCUCUCUCAGGCAUUU-3′ for DR5L (1), 5′-UGUGCUUUGUACCUGAUUCUU-3′ for DR5L (2), 5′-UAUGAUGCCUGAUUCUUUGUG-3′ for DR5S, 5′-GAUUGGAGAAGGCUGGCUC-3′ for FADD (1), 5′-GAACUCAAGCUGCGUUUAU-3′ for FADD (2), 5′-GGUGCACGUUUCAUCAAUU-3′ for Noxa (1), 5′-GCTACTCAACTCAGGAGATTT-3′ for Noxa (2), 5′-GAUCUGCGAUGGCUGCAAUUU-3′ for p62 (1), 5′-GCAUUGAAGUUGAUAUCGAU-3′ for p62 (2), an On-Target Plus siRNA pool of 4 oligonucleotides for RIPK1, 5′-AACGAGCUGAAGCAGAUGCAG-3′ for TRAIL (1), and 5′-UUGUUUGUCGUUCUUUGUGUU-3′ for TRAIL (2).

### Generation of knockout cell lines via CRISPR-Cas9.

HeLa cells (10^5^ cells/well in 6-well plates) were transfected with 1 μg plasmid plentiCas9-BLAST (Addgene plasmid 52962) by using GenJet, followed 24 h later by selection with blasticidin (6 mg/ml) for 7 days. Blasticidin-resistant cells were then seeded as single cells into 96-well plates. After 3 to 4 weeks, colonies derived from single clones were screened for Cas9 expression by immunoblotting. Targeted single guide RNAs (sgRNAs) were designed with the help of the Broad Institute sgRNA design tool (http://portals.broadinstitute.org/gpp/public/analysis-tools/sgrna-design) and cloned into pLentiguidePuro (Addgene plasmid 52963). HeLa cells stably expressing Cas9, generated as described above, were transfected with plasmids containing sgRNAs of interest by using GenJet and selected with 250 ng/ml puromycin for 7 days. Puromycin-resistant cells were seeded as single colonies into 96-well plates. After 3 to 4 weeks, colonies derived from single clones were screened for the expression of proteins of interest by immunoblotting. Sequences are GCGGGGAGGATTGAACCACG for DR4 sgRNA 1, AAGTGTGGGGCTCTTCCGCG for DR4 sgRNA 2, CCAGGACCCAGGGAGGCGCG for DR5 sgRNA 1, CCTAGCTCCCCAGCAGAGAG for DR5 sgRNA 2, GAGGCATAGGAACTTGAGCT for FADD sgRNA 1, GCTCCAGCAGCATGGAGAAG for FADD sgRNA 2, CCAGCTGGACAGTGTCCCGA for CHOP sgRNA 1, and AGCACATCTGCAGGATAATG for CHOP sgRNA 2. Controls used for experiments were cells transfected with the empty vector and subjected to the same procedures.

### Cell death analysis.

Cell death analysis was performed quantifying propidium iodide (PI) incorporation by fluorescence-activated cell sorter (FACS) analysis. After treatment, adherent cells and dead cells in suspension were collected by trypsinization and centrifuged at 450 × *g* for 7 min. The cells were then resuspended in a final volume of 300 μl of phosphate-buffered saline (PBS) plus 0.5 μg/ml of PI and analyzed by using a Gallios flow cytometer (Beckman Coulter). Quantification was done by using FlowJo software version 7.6.4. A lactate dehydrogenase (LDH) test (Promega, Fitchburg, WI) was also used to measure cell death. After treatment, 50 μl of conditioned medium was resuspended in reactive solution and incubated for 30 min at 37°C in a 96-well plate. The absorbance at 490 nm was acquired by using a BioTek PowerWave XS microplate spectrophotometer. Cell death of knockout (CRISPR) cell lines was analyzed by counting dead cells under an inverted microscope. A total of 150 cells per well and 3 wells per condition were counted.

### Immunoprecipitation.

After treatment, cells were collected by using immunoprecipitation (IP) buffer (20 mM Tris-HCl [pH 7.5], 137 mM NaCl, 1% Triton X-100, 2 mM EDTA [pH 8]) containing protease inhibitors (Roche, Basel, Switzerland), lysed on ice for 20 min, sonicated, and quantified by using a bicinchoninic acid (BCA) colorimetric kit (Pierce-Thermo Scientific, Waltham, MA) according to the manufacturer's instructions. The Pure Proteome protein G magnetic bead system (Millipore) was used for immunoprecipitation. Twenty-five microliters of magnetic beads under each condition was blocked by incubation with IP buffer including 1% bovine serum albumin (BSA) for 1 h at 4°C under rotation. The beads were then incubated with 1 μg of primary antibodies for 3 to 4 h at 4°C under rotation. This solution was replaced with 1,200 μg of lysates in 1 ml of IP buffer supplemented with protease inhibitors (Roche), and the mixture was incubated overnight at 4°C under rotation. Beads were then washed 3 to 4 times with IP buffer, and proteins were eluted with IP buffer plus 2% SDS and then incubated with Laemmli buffer (with freshly added 2-mercaptoethanol) and warmed at 95°C for 10 min before being loaded onto an SDS-PAGE gel. Inputs were total lysates and represent 5% of the immunoprecipitated fraction. Primary antibodies used for IP were anti-human caspase-8 p18 (C-20; Santa Cruz, Dallas, TX) and anti-p62 (catalog number BML-PW9860; Enzo, Farmingdale, NY). Anti-glutathione *S*-transferase (anti-GST) (rabbit IgG) and anti-green fluorescent protein (anti-GFP) (mouse IgG), used as isotype controls, were obtained from Rockland (Limerick, PA). Horseradish peroxidase (HRP)-conjugated Clean-Blot IP detection reagent (Thermo Scientific) was used as a secondary antibody for Western blotting of p62.

### Western blotting.

After treatments, cells were collected by trypsinization, mixed with floating cells, washed with PBS, and centrifuged at 500 × *g*. The pellet was resuspended in radioimmunoprecipitation assay (RIPA) buffer (Thermo Scientific) supplemented with protease inhibitors (Roche) and phosphatase inhibitors (Roche). The lysates were then sonicated and quantified by using BCA. Forty micrograms of proteins under each condition was prepared in a 40-μl final volume of 4× Laemmli buffer (63 mM Tris-HCl, 10% glycerol, 2% SDS, 0.01% bromophenol blue, 5% 2-mercaptoethanol) and warmed at 95°C for 10 min. The proteins were loaded onto an SDS-PAGE gel (Mini-protean; Bio-Rad, Hercules, CA) and run at 120 V for 1 h 30 min. The proteins were then transferred to an Immobilon-FL polyvinylidene difluoride (PVDF) membrane (Merck Chemicals, Darmstadt, Germany) using the Trans-Blot SD (Bio-Rad) semidry system for 1 h at 200 mA. The transfer of the proteins was checked by Ponceau S staining (Sigma-Aldrich). Membranes were blocked with 5% nonfat dry milk in Tween–Tris-buffered saline (TBS) (TTBS) or in Odyssey blocking solution (Li-Cor Biosciences, Lincoln, NE) for 1 h at room temperature. The membranes were then incubated with primary antibodies generally diluted 1:1,000 in blocking solution for 1 to 2 h at room temperature or overnight at 4°C with agitation. The next day, the membranes were incubated with HRP-conjugated secondary antibodies diluted 1:5,000 in blocking buffer or with fluorescent secondary antibodies diluted 1:15,000 in a 1:1 solution of TBS-Odyssey blocking buffer for 1 h at room temperature. After 3 washes in TTBS, the membranes were developed with an enhanced chemiluminescence (ECL) reaction using a freshly prepared ECL reagent (Promega) or with the Odyssey infrared imaging system. Primary antibodies were antibodies to actin (C-4; ICN), ATF4 (C-20; Santa Cruz), Bid (catalog number 2002; Cell Signaling, Beverly, MA), cIAP1 (catalog number B75-1; Pharmingen, San Diego, CA), cIAP2 (catalog number AF8171; R&D, Minneapolis, MN), caspase-3 (catalog number 9662S; Cell Signaling), caspase-8 p18 (C-20; Santa Cruz), CHOP (catalog number F-168; Santa Cruz), DR4 (catalog number B-N28 [Diaclone, Besançon, France] or D9S1R [Cell Signaling]) for the data shown in Fig. 9F, DR5 (catalog number D4E9; Cell Signaling), FADD (catalog number H-10 [Santa Cruz] or 610400 [BD Transduction Laboratories]) for the data shown in Fig. 6F, GRP78 (N-20; Santa Cruz), PARP (catalog number 9542S; Cell Signaling), p62 (catalog number BML-PW9860; Enzo), RIPK1 (catalog number C38; Pharmingen), and TRAIL/TNFSF10 (catalog number 55B709.3; Novus Bio). HRP-conjugated secondary antibodies were anti-rabbit and anti-mouse antibodies from Zymax and anti-goat antibody from Rockland. Secondary anti-mouse, anti-rabbit, or anti-goat antibodies (IRDye 800CW donkey anti-rabbit, IRDye 680LT donkey anti-mouse, and IRDye 680CW donkey anti-goat antibodies) were obtained from Li-Cor Biosciences.

### Immunocytochemistry.

Cells were plated in 12-well plates (BD) on 12-mm round sterile coverslips precoated with a 0.1% poly-l-lysine solution (Sigma) at 37°C. Twenty-four hours later, cells were treated at a confluence of 70 to 80% without glucose for 24 h. Cells were then fixed with a PBS–4% paraformaldehyde solution (Merck) for 20 min at room temperature. Cells were washed 3 times with PBS for 5 min each and then incubated with blocking buffer (0.05% Triton, 3% BSA in PBS) for 1 h, with shaking, at room temperature. The cells were then incubated with primary antibodies diluted 1:200 in blocking buffer at 4°C overnight in a humid covered dish. The coverslips were washed 3 times with PBS for 5 min each and incubated for 1 h with secondary antibodies diluted 1:400 in blocking buffer at room temperature. Cells were then washed 3 times with PBS and once with 4′,6-diamidino-2-phenylindole (DAPI) diluted 1:10,000 in PBS for 10 min at room temperature. The coverslips were then mounted by using 3 μl of Vectashield solution (Vector Laboratories, Burlingame, CA) and allowed to dry for at least 1 day. Photographs at different zooms were acquired by using the Suite Advanced Fluorescence software application (2.6.0.7266; LAS AF) using a Leica TSC SP5 spectral confocal microscope with an HCX PLAPO lambda blue 63×, 1.4-numerical-aperture objective. Colocalization analysis was done by using the colocalization plug-in of Fiji/Image J software, measuring three images per experiment (∼80 cells). The graphs show the averages and the standard deviations of data from at least three independent experiments. Primary antibodies used for immunocytochemistry were anticalnexin (catalog number AF18; Abcam), anti-human caspase-8 (C-20; Santa Cruz), anti-DR5 (catalog number D4E9; Cell Signaling), anti-FADD (catalog number H-181; Santa Cruz), anti-GM130 (catalog number 35; BD Pharmingen), and anti-p62 (catalog number BML-PW9860; Enzo). Secondary antibodies were rabbit Alexa Fluor 568 (Life Technologies, Waltham, MA)-conjugated, mouse Alexa Fluor 647 (Life Technologies)-conjugated, and goat Alexa Fluor 488 (Life Technologies)-conjugated antibodies.

### qPCR analysis.

Cells were collected at room temperature by trypsinization, washed once with PBS, and centrifuged for 5 min at 300 × *g*. RNA was extracted from the pellets by using the RNeasy minikit (Qiagen, Valencia, CA) according to the manufacturer's instructions. One microgram of RNA under each condition was retrotranscribed to cDNA by using the High-Capacity cDNA reverse transcription kit from Applied Biosystems (Waltham, MA). qPCR analysis was performed by using LightCycler 480 SYBR green I Master solution (Roche) starting with 10 ng of cDNA per reaction mixture. Amplification was performed with a LightCycler 96-well plate (Roche) by using the following protocol: a preincubation step of 1 cycle at 95°C, amplification for 45 cycles at 95°C (primer-dependent temperature) and 72°C, a melting curve of 1 cycle at 95°C, 65°C, and 97°C; and cooling for 1 cycle at 40°C. The primers used for analyses were hTRAIL-R1 (DR4) (forward primer GCTGTGCTGATTGTCTGTTG and reverse primer TCGTTGTGAGCATTGTCCTC) and hTRAIL-R2 (DR5) (forward primer TGAGACCTTTCAGCTTCTGC and reverse primer ATCGTGAGTATCTTGCAGCC). The housekeeping gene used for analysis was the RPL32 gene (forward primer AACGTCAAGGAGCTGGAAG and reverse primer GGGTTGGTGACTCTGATGG). Results were further normalized to the values for control treatment.

### RT-PCR analysis of spliced XBP1.

Total RNA was isolated by using the RNeasy minikit (Qiagen) according to the manufacturer's instructions. One microgram of RNA under each condition was retrotranscribed at cDNA by using the High-Capacity cDNA reverse transcription (RT) kit from Applied Biosystems. Amplification was performed with a 2720 thermal cycler (Applied Biosystems, Waltham, MA) by using the following protocol: 95°C for 5 min, 95°C for 1 min, 55°C for 1 min, and 72°C for 1 min for 34 cycles and 72°C for 1 min, 72°C for 5 min, and 16°C to the end. The primers used for analysis were forward primer TTACGAGAGAAAACTCATGGCC and reverse primer GGGTCCAAGTTGTCCAGAATGC. PCR products were analyzed on an 8% acrylamide gel.

### Statistics.

Error bars represent the standard errors of the means (SEM). The significance of data was measured by using two-tailed, paired Student's *t* test. Significant differences are marked as indicated in the figure legends.
